# Editorial: New trends in cardiovascular development, evolution and disease

**DOI:** 10.3389/fcell.2024.1392713

**Published:** 2024-04-12

**Authors:** Ugo Coppola, Diego Franco

**Affiliations:** ^1^ Molecular Cardiovascular Biology Division and Heart Institute, Cincinnati Children’s Hospital Medical Center, Cincinnati, OH, United States; ^2^ Cardiovascular Development Group, Department of Experimental Biology, Faculty of Experimental Sciences, University of Jaen, Jaen, Spain

**Keywords:** cardiovascular development, CHDs, heart evolution, postnatal heart biology, cardiac bioinformatics, ECM, extracellular matrix

The heart represents the main conduit of the circulatory system, with the primary role of pumping the blood through the pulmonary and all the systems of the body (Holmes, 1975; Standring, 2015). Remarkably, multiple scientific contributions demonstrated that the hearts of the amniotic vertebrates share a common plan during their development, with the involvement of conserved transcriptional mechanisms controlling the formation of the cardiac chambers (atria, ventricles) and the associated structures (Fishman and Olson, 1997; Olson, 2006).

The heart is a dynamic multicellular organ, formed by heart-muscle cells, blood vessels, inflammatory cells, fibroblasts, and extracellular tissues. Comprehending the normal heart functioning and development is a fundamental challenge because cardiac dysfunctions can lead to congenital heart defects (CHDs), as well as heart failure and life-threatening arrhythmias. Thus, understanding the molecular etiologies of cardiac pathologies is essential to define novel targets to counteract or heal CHDs. The heart of vertebrates is the product of millions of years of incessant evolution and dainty changes (Olson, 2006), originating by the heart of invertebrate chordates (Cephalochordates and Urochordates) (Stephenson et al., 2017). Henceforth, our Research Topic encompasses two reviews and four research articles, focusing on multiple aspects of cardiac development, evolution and diseases in vertebrates. The articles present in this Research Topic fall into innovative categories, such as cardiac evo-devo, postnatal heart biology, cardiac bioinformatics.

Cardiac evo-devo biology represents a relatively unexplored field for cardiovascular biologists, with two Research Topic contributions pointing at distinct cardiac evolutionary questions. Interestingly, through an integrated approach of phylogeny, transcriptomics, lineage tracing and genetic studies, Demoya et al. described a novel conserved mechanism in vertebrate heart development. They highlighted the evolutionary conserved role of *sap130a* in ventricle formation. Specifically, their data support an elegant model whereby the Sap130a/Sin3a/Hdac complex regulates ventricular differentiation in vertebrates. A second study from Graham et al. reviewed the features that control the formation of arteries of the pharyngeal arches. Specifically, they described the differences and the analogies of pharyngeal arches and their associated arteries among all classes of vertebrates. Furthermore, they use the arteries to propose a new classification of vertebrate pharyngeal arches, which will reflect the vessel’s positions. Moreover, they report how defects in arch artery biogenesis can lead to lethal cardiovascular malformations, such as cervical origin of arteries and vascular rings.

A further significant aspect of cardiovascular biology is the mammalian postnatal period, with the heart undergoing relevant changes due to increase in circulation activity. Here, three studies focused on the cellular modifications that occur during postnatal period in mammals. Santamaria et al. measured the effects of capillary pruning on the maturation of postnatal cardiomyocytes. In particular, they utilized novel tools to visualize the 3D coronary microvasculature of the postnatal mouse heart and tested two vasodilators (losartan and prazosin). Through a combination of different approaches such as imaging, proteomics and *in vivo* functional assays, they described changes in coronary network functionalities, suggesting new strategies to heal microvasculature damages. Furthermore, Uscategui Calderon et al. reviewed the molecular mechanisms underlying the crosstalk between cardiomyocytes and fibroblasts within mammalian postnatal heart. Specifically, they highlighted the importance in the cardiomyocyte-fibroblast interactions of extracellular matrix (ECM) components. Their review reports multiple aspects of ECM remodeling that control fundamental steps of postnatal cardiac development, indicating potentially interesting therapeutic targets. Notably, Lintao et al. characterized for the first time a cellular subpopulation in mouse maternal hearts. Using a *Cre* reporter mouse model, they described the composition of microchimeric immune cells in the maternal heart microenvironment during normal and pathologic pregnancies. Recently, bioinformatics and machine data learning offered various contributions to cardiovascular biology. Intriguingly, Tong and Sun integrated them to identify new markers for atrial fibrillation (AF), the most common of arrhythmia in humans. On the basis of previously described datasets of differentially expressed genes (DEGs) between AF and sinus rhythm samples, they explored the interrelation existing amongst hub genes, immune microenvironment and immune regulation. Of note, their integrated approach led to identify *HIF1AN* and *MPV17* as key hub genes, which are implicated in mitochondrial dysfunction and oxidative stress.

In conclusion, our Research Topic covered different aspects of heart biology in vertebrates, providing innovative information for the community of the cardiovascular researchers and encompassing distinct topics that included cardiac development, evolution and diseases.
